# Quantitative analysis of proteins which are members of the same protein complex but cause locus heterogeneity in disease

**DOI:** 10.1038/s41598-020-66836-7

**Published:** 2020-06-26

**Authors:** Alessio Gamba, Mario Salmona, Gianfranco Bazzoni

**Affiliations:** Department of Biochemistry and Molecular Pharmacology Istituto di Ricerche Farmacologiche Mario Negri IRCCS Via Mario Negri 2, I-20156 Milano, Italy

**Keywords:** Computational biology and bioinformatics, Systems biology, Medical genetics

## Abstract

It is still largely unknown how mutations in different genes cause similar diseases – a condition known as locus heterogeneity. A likely explanation is that the different proteins encoded by the locus heterogeneity genes participate in the same biological function and, specifically, that they belong to the same protein complex. Here we report that, in up to 30% of the instances of locus heterogeneity, the disease-causing proteins are indeed members of the same protein complex. Moreover, we observed that, in many instances, the diseases and protein complexes only partially intersect. Among the possible explanations, we surmised that some genes that encode proteins in the complex have not yet been reported as causing disease and are therefore candidate disease genes. Mutations of known human disease genes and murine orthologs of candidate disease genes that encode proteins in the same protein complex do in fact often cause similar phenotypes in humans and mice. Furthermore, we found that the disease-complex intersection is not only incomplete but also non-univocal, with many examples of one disease intersecting more than one protein complex or one protein complex intersecting more than one disease. These limits notwithstanding, this study shows that action on proteins in the same complex is a widespread pathogenic mechanism underlying numerous instances of locus heterogeneity.

## Introduction

Gene mutations exert their primary and direct effects at the molecular level, by affecting the structure and function of the gene products that the genes encode. In turn, the abnormal disease gene products – mostly proteins – predictably affect various biological structures and functions at increasingly higher levels of anatomical organization, such as organelles, cells, tissues, organs and systems. Finally, the ultimate indirect consequences of an initial gene mutation are the clinical manifestations of disease, i.e. the disease phenotypes observed at the level of the whole organism. Thus identifying the sequence of biological processes that mechanistically link the initial molecular dysfunction to the ultimate clinical phenotypes can be expected to lead to deeper understanding of genetic diseases.

It is important to bear in mind that the vast majority of proteins do not work in isolation. They work together with other proteins, often in protein complexes (PC), which are biochemically cohesive and functionally homogeneous assemblies^1^. A global proteomic analysis identified more than 600 PC associated with core biological functions^2^. A mutation in a given disease-causing gene is therefore likely to affect – even disrupt – the overall structure and function of the PC containing the protein encoded by the mutated gene^3^. This hypothesis is borne out by studies of protein-protein interactions, showing that physical interactions are frequent among proteins responsible for similar phenotypes^4–8^. Lastly, the association between PC and diseases has been confirmed by integrative ‘phenome-interactome’ analyses^9^ and other reports^10–13^, which identified many disease modules, i.e. clusters of disease proteins (and their interacting proteins) that cause similar disease phenotypes.

The present study focuses on locus heterogeneity (LH), the condition whereby mutations in different genes cause diseases which, clinically, display remarkably similar, or even identical, phenotypes^14^. While most cases of LH are causally linked to mutations in just a few genes (usually two or three), less frequent cases are caused by mutations in many more genes. For instance, the more than 50 disease proteins causing *Retinitis pigmentosa*, a progressive degeneration of the retina, support the notion that the same pathological phenotype can result from the dysfunction of dozens of diverse (and sometimes apparently unrelated) proteins^15^.

**Figure 1:**
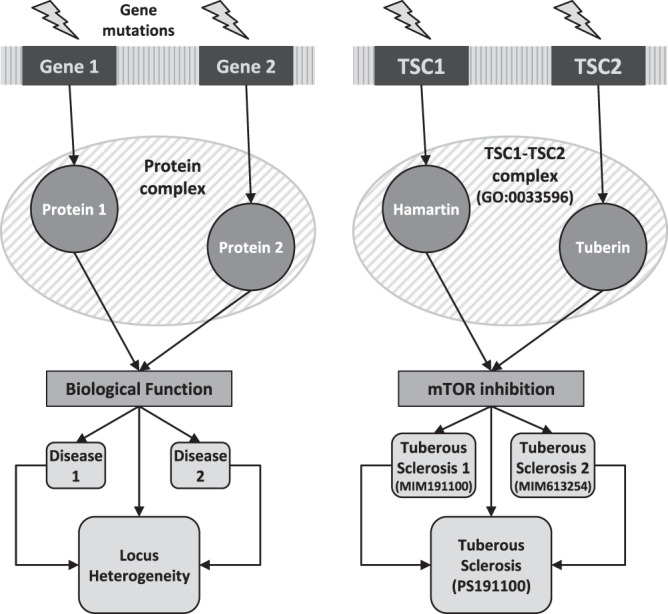
*Disruption of different proteins within the same PC as general mechanism of LH*. The working hypothesis is shown schematically (*left*). Mutations of either Gene 1 or Gene 2 affect the corresponding proteins (Protein 1 or Protein 2). If the proteins are members of the same PC, then the (molecularly different but clinically similar) Disease 1 and Disease 2 (an instance of LH) are the result of alternative means of disrupting the same PC, thereby interfering with the PC’s biological function. In the example shown (*right*), mutations of either the *TSC1* or *TSC2* gene affect the proteins *Hamartin* or *Tuberin*, which are members of the *TSC1-TSC2 complex*. Thus the resulting diseases *Tuberous sclerosis-1* or *-2* (both in the PS *Tuberous sclerosis*) can be viewed as alternative means of interfering with the function that is normally carried out by the PC, namely inhibition of mTOR signaling.

Thus, within the conceptual framework of the well-established link between diseases and PC, we surmised that the increasing knowledge on the PC could shed light on the basic mechanisms of LH. One can envision that several cases of LH might be due to mutations affecting different members of the same PC (Fig. 1, *left panel*). In other terms, the mutations of different genes that result in the same disease (i.e. in LH) could be explained mechanistically as alternative ways of affecting the same cellular machinery (the PC).

To analyze the LH-PC connection, we drew on the breadth of coverage and accuracy of annotation of the Online Mendelian Inheritance in Man (OMIM) database^16^. We employed two working definitions of LH. First, we defined as LH those (relatively few) instances in OMIM in which two (or more) diseases have the same identifier (hereafter, ‘disease-based LH’). For instance, *Colon cancer, somatic* and *Colorectal cancer, somatic*, in spite of the different names, are both annotated with the same OMIM identifier, 114500.

Second, to broaden our search, we added as definition of LH those (more numerous) instances in OMIM in which two or more diseases are included in the same phenotypic series (PS), in spite of having different names and identifiers (hereafter, ‘PS-based LH’). The PS are a recently added feature of OMIM and designate clusters of clinically similar diseases^16^. For instance, seven different types of hereditary colorectal cancer, which have seven different disease identifiers in OMIM, have been assigned to the *Colorectal cancer, hereditary nonpolyposis* PS and have the same PS identifier, *PS1204*3*5*.

Finally, we counted the occurrences of disease- and PS-based LH in which the disease-causing proteins were also members of the same PC. In the example shown in Fig. 1 (*right panel*), both *TSC1* and *TSC2* genes (encoding the *Hamartin* and *Tuberin* proteins, respectively) annotate two diseases (*Tuberous sclerosis-1* and *-2*), which belong to the same PS (*Tuberous sclerosis*) and, accordingly, have the same PS identifier, *PS191100*, even though they have different disease identifiers (*MIM 191100* and *MIM 61*3*254*).

## Results

### Disease-PC intersection in disease-based LH

**Figure 2:**
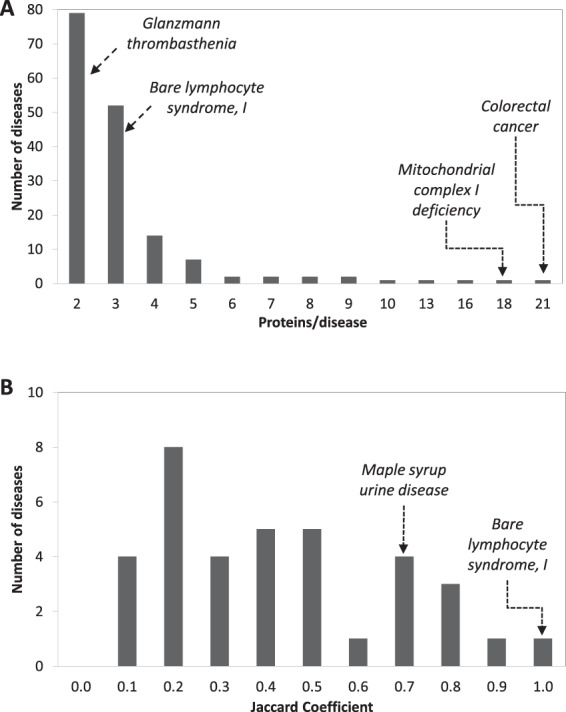
*Disease-PC intersection (disease-based LH)*. (**A**) The 165 instances of disease-based LH were divided according to the number of disease proteins that cause the same disease. For instance, 79 diseases (including *Glanzmann thrombasthenia*) are caused by mutations in two different genes. Other examples of diseases are indicated (*dashed arrows*). (**B**) The intersection of LH-associated diseases with PC was divided into discrete classes of increasing JC. When a disease intersected more than one PC, only the JC_MAX_ was taken into account (i.e. the highest JC among the disease-PC pairs for that disease).

Under the disease-based definition of LH, we first searched OMIM for the gene-disease annotations in which more than one gene annotates the same disease. We found that 4.1% of the diseases (165 out of 4,011) are instances of LH. The average number of encoded proteins per disease is 3.3 ± 2.6, but there is wide variability (Fig. 2A). While the majority of LH diseases are linked to just two (e.g. *Glanzmann thrombasthenia*) or three proteins (e.g. *Bare lymphocyte syndrome, type I*), a few diseases are linked to more; these include *Mitochondrial complex I deficiency* (18 proteins) and *Colorectal cancer* (21 proteins).

Next, we counted the intersections between this set of LH-diseases and the PC (according to the definition of PC in Gene Ontology; see Methods). We compared the 165 LH diseases with 575 PC. These PC comprise 3,291 proteins (on average 7.8 ± 11.2 proteins per PC). We found that 21.8% of the LH-diseases (36 out of 165) share at least two proteins with at least one PC. To quantify the intersection, we calculated a Jaccard Coefficient (JC), which ranges from 0 (no intersection) to 1 (full intersection). When a disease intersected with more than one PC, only the maximum JC was taken into account. The average JC is 0.40 ± 0.25 (median 0.33; Fig. 2B). One disease, *Bare lymphocyte syndrome type I*, has a full intersection with the *TAP complex*, because three proteins (*TAP1*, *TAP2* and *TAPBP*) are the proteins causing the disease and the members of the PC at the same time. Thus about 4% of the diseases are instances of disease-based LH and about one fifth share at least two proteins with a PC.

### PS-PC intersection in PS-based LH

**Figure 3:**
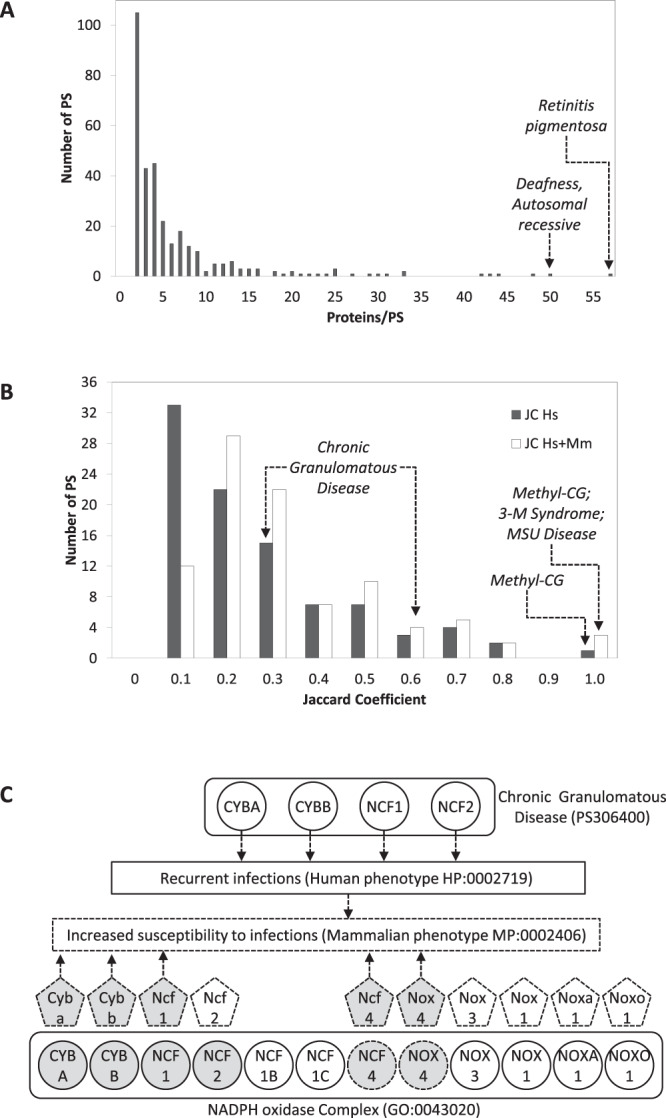
*Disease-PC intersection (PS-based LH)*. (**A**) The 319 PS examined were divided according to the number of disease proteins per PS. (**B**) The PS-PC intersections were divided into discrete classes of increasing JC. *Black bars* indicate the human disease proteins alone, and *white bars* refer to the union of the human disease proteins plus the murine orthologs of the human non-disease proteins that cause similar phenotypes in mice and humans. (**C**) The example of *Chronic granulomatous disease* (see Results for details).

Under the PS-based definition of LH, we first searched for the gene-disease annotations in which more genes annotate diseases within the same PS. We selected 319 PS containing at least two diseases with a known molecular basis. These PS comprise 2,079 diseases and 1,758 disease proteins. On average, a PS contains 6.7 ± 8.3 diseases and 6.6 ± 8.1 proteins, though a few PS (e.g. *Retinitis pigmentosa*) comprise many more diseases and proteins (Fig. 3A).

Then we searched for the PS-PC intersections, by comparing the 319 PS with the 575 PC. We found that 29.8% of the PS (95 out of 319) shared at least two proteins with the same PC. Conversely, 17.4% of the PC (100 out of 575) shared at least two proteins with the same PS. The average JC was 0.23 ± 0.21 (median 0.17; Fig. 3B, *black bars*).

Then to assess the significance of the PS-PC intersections, we ran a series of control simulations (each control was run 100,000 times). First, we randomized either the PS or the PC. To randomize the PS we distributed the 1,758 disease proteins randomly among 319 pseudo-PS (i.e. clusters of proteins the same size as the real, non-randomized, PS); only 10.4 ± 2.7 pseudo-PS (compared with 95 real PS) shared at least two proteins with the same real PC (*Z* = 30.8). Then, to randomize the PC, we distributed the 3,291 proteins of the PC randomly among 575 pseudo-PC (clusters of proteins the same size as the real PC); only 10.3 ± 2.7 pseudo-PC (compared with 100 real PC) shared at least two proteins with the same real PS (*Z* = 32.6).

Second, we analyzed the non-LH proteins (i.e. disease proteins not associated with LH), distributing 1,062 non-LH proteins randomly among the 319 pseudo-PS. Only 5.5 ± 2.1 pseudo-PS composed of non-LH proteins (compared with 95 real PS composed of LH-proteins) shared at least two proteins with the same real PC (*Z* = 41.9). Third, we examined all the possible pairwise combinations of the 1,758 disease proteins. The probability of two proteins (in each pair) causing two different diseases within the same PS was 26 times higher when both proteins belonged to the same PC (352 pairs), compared with randomly paired proteins (13.5 ± 3.6 pairs; *Z* = 94.0). All these control experiments indicate that the PS-PC relations observed are more frequent than would be expected from chance alone.

Concerning the variability of the JC, one should consider that the coefficient correlates not only directly with the PS-PC intersection (i.e. the number of proteins shared by PS and PC) but also inversely with the PS-PC union (i.e. their total number of proteins). As the PC vary widely in size (from 2 to 116 proteins per PC, with a median of 13), the JC vary accordingly and larger PC tend to have smaller JC. The average JC was 0.31 ± 0.22 for PC equal to or smaller than the median (≤13 proteins), whereas it was only 0.08 ± 0.65 for bigger PC ( > 13 proteins). The effect of the size of the PC on the JC is illustrated by the *Atrial fibrillation* PS and its two intersecting PC, i.e. the voltage-gated channels for sodium and potassium. Although the same number of proteins (five proteins) account for the intersection of the PS with either of the PC, the two PC differ greatly in size (one 14 and the other 88 proteins). Consequently the JC for the two PS-PC also differ greatly (0.238 and 0.053, respectively), in spite of the identical intersection. With this caveat, we conclude that, like the disease-based LH, the PS-based LH too is significantly related with the PC.

### Partial PS-PC relations: murine orthologs of ‘non-disease proteins’ PC members as candidate disease genes

Besides the size of the PC (and the PS), there are other possible reasons for the low JC. One might be our lack of knowledge about the ‘non-disease proteins’ – the proteins of the PC that have not yet been reported as disease proteins in humans. We thought, however, that knowledge might be available about mutations that affect murine orthologs of the human non-disease proteins and cause phenotypes in mice similar to the phenotypes that the disease proteins of the same PC cause in humans.

Thus, for each PC, we considered as presumptive disease proteins any human non-disease proteins with known murine-to-human phenotypic resemblance. Then we calculated a new JC. This way, the JC actually increases (Supplementary Table S1). First, in the initial set of 319 PS, the number of PS intersecting a PC rises from 95 (29.8%; human disease proteins only) to 142 (44.5%; human disease proteins plus human non-disease proteins that are orthologs of murine disease proteins). Second, in the set of 95 PS, inclusion of these orthologs raises the average JC from 0.23 ± 0.21 to 0.31 ± 0.23 (and the median JC from 0.17 to 0.23; Fig. 3B, *white bars*).

For instance, mutations in four human disease proteins (CYBA, CYBB, NCF1 and NCF2; Fig. 3C, *circles*) cause four similar diseases that belong to the PS *Chronic Granulomatous Disease*. As these four proteins are also members of the 12-protein PC *NADPH oxidase complex*, the JC is 0.33. However, mutating Ncf4 and Nox4, two murine orthologs (*pentagons*) of the non-disease proteins (but PC members) NCF4 and NOX4, causes the phenotype *Increased susceptibility to infection* in mouse. This latter resembles the phenotype *Recurrent infections* in humans, which is caused by mutations in the four disease proteins of the PS. Thus, including NCF4 and NOX4 as presumptive disease proteins increases the PS-PC intersection from 4 to 6 proteins and the JC from 0.33 to 0.60.

### Partial PS-PC relations: gain-of-function and lethal mutations

Among the other reasons for partial PS-PC intersections, gain-of-function mutations may cause diseases without necessarily affecting the localization of a given disease proteins to a PC (if any). Hence potential sets of LH-associated proteins might not be found in the same PC (or in any PC at all). Supplementary Table S2 lists the PS-PC pairs in which variable numbers of gain-of-function mutations cause diseases that belong to the PS, whereas the corresponding proteins do not belong to the PC (and thus do not contribute to the PS-PC intersection).

Furthermore, some members of a PC might be essential proteins. Therefore, if loss-of-function mutations of these proteins cause pre-birth lethality, they might not be listed as disease-causing proteins in OMIM. Supplementary Table S3 reports the PC that contain at least one protein whose ortholog causes pre-birth lethality at various stages of intra-uterine development (embryonic, fetal or prenatal) in the mouse.

### Non-univocal PS-PC relations: redundancy of the PC?

All these data indicate that a considerable number of PS do intersect the PC. However, only 37 (out of 95) PS have a univocal 1:1 PS-PC relation. In the remaining 58 PS there were two different types of non-univocal relation.

In the former type of non-univocal PS-PC relation, the same PS intersects more than one PC (from 2 to 5 PC per PS). However, closer analysis indicates that most instances involve different sorts of PC redundancy. First, some PC are in a hierarchical parent-to-child relation. For instance, the PS *Cornelia de Lange syndrome* intersects both the *Meiotic cohesin complex* and its parent *Cohesin complex*. Second, some PC are children of another PC. For instance, the PS *Hermanski-Pudlak syndrome* intersects three PC (*Bloc-1*, *Bloc-2* and *Bloc-*3 *complex*), which are all children of *Bloc complex*. Third, some other PC share numerous proteins, suggesting that the PC are mere variants of the same biochemical entity. For instance, the PS *Epileptic encephalopathy, early infantile* intersects both *Chloride channel complex* and the *GABA-A receptor complex*, even though the two PC share 18 proteins (and the latter is fully enclosed in the former).

**Figure 4:**
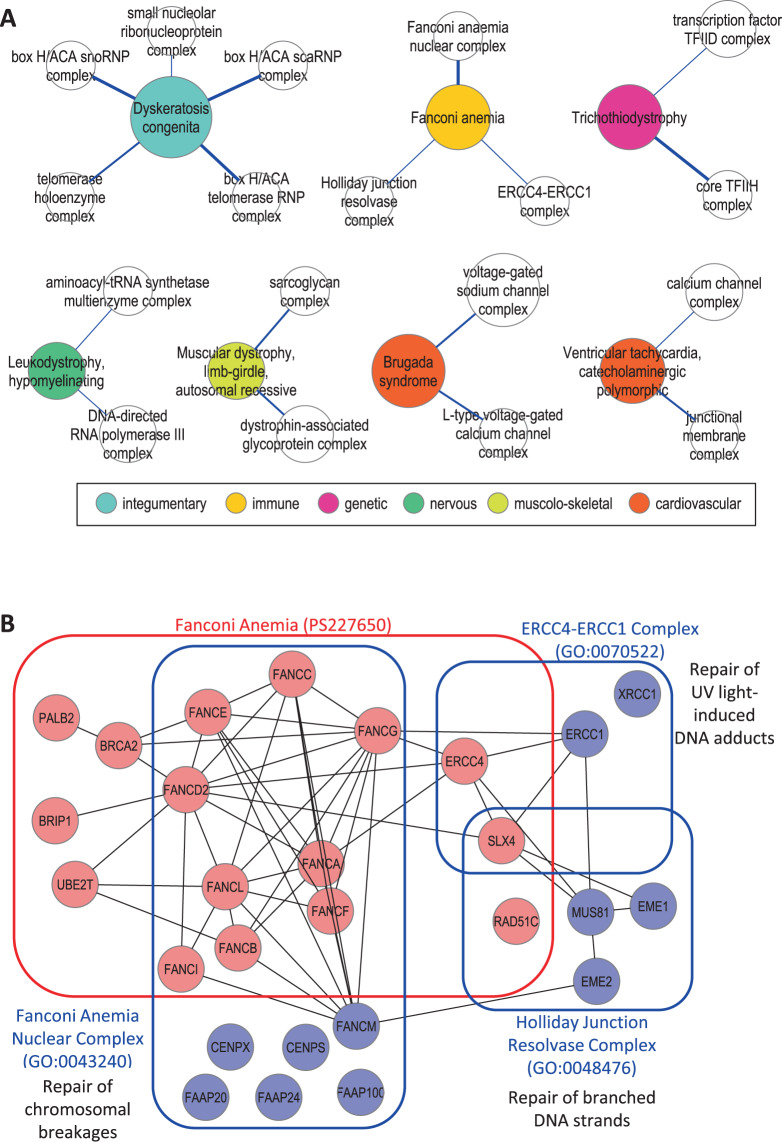
*One-to-many PS-PC relationships*. (**A**) The nodes represent PS (*colored circles*) and PC (*white circles*), while the edges linking the nodes represent the PS-PC relations, the *edge width* being proportional to the JC. The *colors* of the *circles* indicate the classification of the diseases within that PS (according to Disease Ontology; *inset*). For simplicity’s sake, only PS-PC intersections with JC ≥ 0.1 are shown in the Figure. (**B**) The proteins shown account for the intersection of the PS *Fanconi anemia* (*red rectangle*) with the three PC (*blue rectangles*). The proteins are the disease proteins (*pink*) that cause the diseases in the PS and the non-disease proteins (*light blue*) that are members of the PC, while the edges indicate protein-protein interactions. The graphs were drawn with Cytoscape 2.8.3 and yEd 3.15 (available at cytoscape.org and yworks.com).

We therefore focused on the ‘one-to-many’ PS-PC intersections that are unlikely to be due to PC redundancy (Fig. 4A). For instance, the PS *Fanconi anemia* intersects three PC, namely *Fanconi anemia nuclear complex*, *ERCC4-ERCC1 complex* and *Holliday junction resolvase complex* (Fig. 4B). Although biochemically unrelated, these three PC converge towards the same biological function – DNA repair, even though each one specifically repairs a distinct type of DNA damage, respectively chromosomal breakages, ultraviolet light-induced DNA adducts and branched DNA strands

### Non-univocal PS-PC relations: redundancy of the PS?

**Figure 5:**
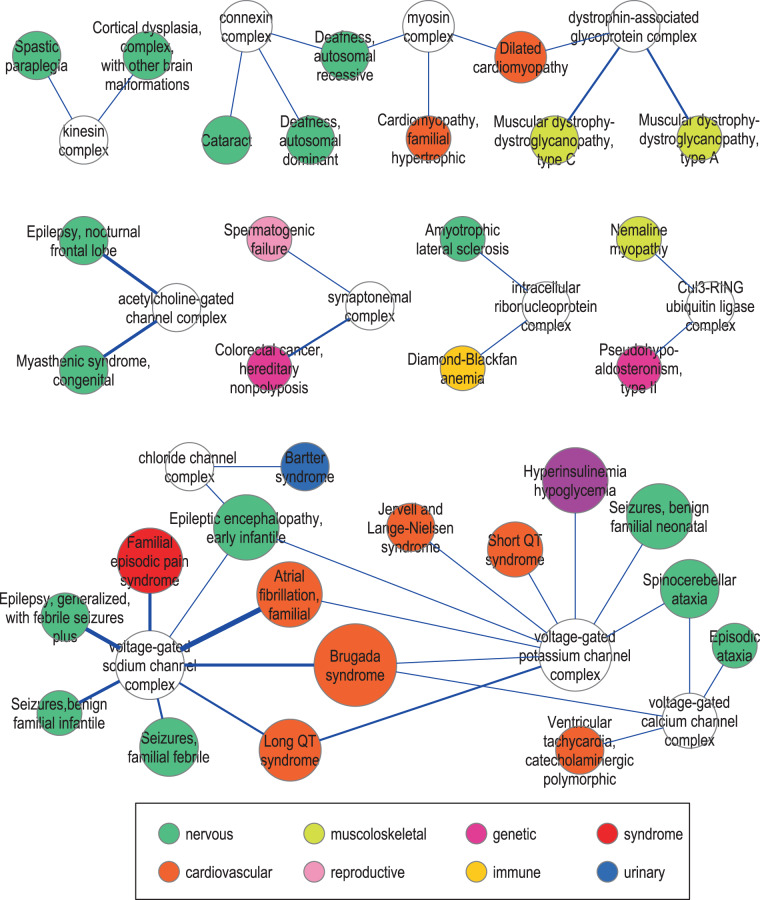
*Many-to-one PS-PC relationships*. The nodes represent PS (*colored circles*) and PC (*white circles*). The *size* of the nodes is proportional to the number of links, and the *colors* indicate the classification of the diseases within that PS (according to Disease Ontology; *inset*). The edges represent the PS-PC relations (*blue lines*), the *width* of the edge being proportional to the strength of the link (i.e. the JC). The graph was drawn with Cytoscape 2.8.3 and yEd 3.15 (available at cytoscape.org and yworks.com).

In the latter type of non-univocal PS-PC relations, more than one PS intersects the same PC. However, most are instances of PS redundancy due to allele heterogeneity, whereby the same disease proteins causes more diseases, and consequently more PS. Here we focused on the ‘many-to-one’ PS-PC intersections that are unlikely to be due to allele heterogeneity (Fig. 5). At first sight, in some of these non-univocal relations the various PS intersecting the same PC are clinically similar; examples are the PS *Spastic paraplegia* and *Cortical dysplasia* that intersect the *Kinesin complex*. Similarly, five syndromic PS, all characterized by cardiac arrhythmias (*Brugada*, *Long QT*, *Atrial fibrillation familial, Short QT*, as well as *Jervell and Lange-Nielsen Syndrome*), intersect the same PC (the *Voltage-gated potassium channel*).

## Discussion

Seeking to clarify better the mechanisms of LH, we examined the hypothesis that the mutations of most LH-related genes, while affecting different proteins, might interfere with the same function or structure, because these genes encode members of the same PC (Fig. 1). A PC is a stable molecular assembly that supports a biological function and/or a subcellular structure. We consulted detailed databases in order to identify both LH (OMIM) and PC (Gene Ontology). We focused initially on diseases that have the same identifier (*disease-based LH*). The set, however, is limited, due to the OMIM commitment to provide a detailed molecular classification of the diseases. This means that clinically similar, but molecularly different, diseases are usually assigned different identifiers (Fig. 2). To get round this obstacle, we extended our search to a wider set of diseases that, in spite of having different identifiers, belong to the same PS (*PS-based LH*). The high degree of clinical similarity among diseases within the same PS, which only differ in the molecular identity of the mutated genes^16^, supports our decision to consider these diseases as proper instances of LH (Fig. 3). The results reported here lend support to the working hypothesis with regard to both definitions of LH, as we did detect an intersection of at least two LH-proteins with a PC in about 22% and 30% instances of disease- and PS-based LH, respectively.

However, besides supporting the hypothesis, our results also indicate that the extent of the PS-PC intersection (as quantified by the JC) is variable and that, in some intersections the JC is only small. A likely explanation is that many occurrences of disease similarity might depend on protein-protein interactions outside the PC. Nevertheless, we believe that, even when the JC is low the PS-PC intersection can be biologically important. On one hand, the JC is low if the PS-PC intersection (the numerator of the JC) is low, i.e. when just a few proteins are shared by the PS and PC. However, the JC is also low when the PS and/or the PC (the denominator) is large – even when the PS-PC intersection *per se* is not negligible. For instance, all three disease proteins in the PS *Short QT syndrome* are members of the PC *Voltage-gated potassium channel complex* and the syndrome is indeed regarded as a ‘channelopathy’^17^. However, since the PC encompasses almost 90 proteins, the JC is just 0.034.

Another possible reason for narrow PS-PC intersections (and thus small JC) is the presence in the PC of non-disease proteins, i.e. proteins that have not yet been reported to cause diseases in humans. To address this, we consulted data on the phenotypes of mutant alleles in mice^18^. Fortunately, these phenotypes have been organized in structured vocabularies, facilitating mouse-to-human comparisons^19^. With these datasets, we confirmed that mutations of many murine orthologs of human non-disease proteins (within the PC) do result in abnormal phenotypes. In turn, the phenotypes in mice are often similar to the phenotypes in humans that result from mutations of genes encoding the disease proteins within the same PC. The importance of identifying these genes is two-fold. First, it stresses the significance of the PS-PC intersections. Second, the products of these genes can be regarded as candidate disease proteins for future experimental research and as potential therapeutic/diagnostic targets for clinical use^20^.

Narrow PS-PC intersections could also be due to gain-of-function mutations which, in contrast to the (more common) loss-of function mutations, might affect a biological process without interfering with the protein’s ability to localize to the PC. In addition, the PC themselves are resistant to gain-of-function mutations^21^ and some of these mutations might even result in the assembly of new PC^22^. We found many examples of PS-PC pairs containing at least one gain-of-function protein that does not belong to the PC (alongside loss-of-function proteins that instead do belong to the PC; Table S2). We detected even a few PS that do not intersect any PC at all and that contain more than one gain-of-function protein. For instance, mutations causing over-expression or over-activation of components of the Ras signaling pathway result in six similar diseases within the PS *Noonan Syndrome*, a PS that we have been unable to associate with any PC^23^.

Finally, another possible reason for narrow PS-PC intersections is the presence within a given PC of proteins essential for intra-uterine viability, as documented in Table S3. As their mutations often result in spontaneous abortions, it is likely that many of these proteins are not reported as disease-causing proteins in OMIM. Nevertheless, it should be added that, being critical components of the cell, many PC may have evolved structural robustness and functional redundancy, to accommodate the mutations of their protein components^24^.

Our analysis also highlighted two types of non-univocal PS-PC relation – exceptions to the ‘one PS-one PC’ rule we had expected. While these non-univocal PS-PC relations seemingly weaken the general hypothesis, further analysis indicated that they reflect redundancy of annotations more often than multiplicity of mechanisms.

Concerning the non-univocal ‘one PS-more PC’ relation, the various PC that intersect the same PS are often closely related. The relation can be either semantic (the PC are parent and child) or physical (the PC share many proteins). For instance, the concept that the *GABA-A receptor complex* is indeed a *Chloride channel complex*^25^ provides a simple explanation for the intersection of *Epileptic encephalopathy, early infantile* with both PC. Nonetheless, even excluding these redundancies, a handful of PS-PC relations remain (Fig. 4). Yet even these few exceptions to the ‘one PS-one PC’ rule somehow broaden the sense in which the PC can act as a site of LH. For instance, the PS *Fanconi anemia* intersects three distinct PC. However, far from being functionally unrelated, all three serve the general purpose of repairing DNA, even though each PC repairs a different type of DNA damage. One of the major goals of research in the DNA field is to understand how these PC cooperate to carry out the different forms of repair^26^.

Concerning the non-univocal ‘more PS-one PC’ relation, the relative abundance of PS intersecting the same PC often reflects allele heterogeneity, whereby the same protein causes diseases in more than one PS. This is the case, for instance, of two PS (*Aortic aneurysm, familial thoracic* and *Loeys-Dietz syndrome*) that intersect the PC *Transforming Growth Factor beta-receptor complex*. Nevertheless, we also identified examples of ‘more PS-one PC’ relations that are not explained by allele heterogeneity. Interestingly, these PS resemble each other in their clinical manifestations (for instance, the cardiac arrhythmia-related PS in Fig. 5). Therefore, we propose that these mutations, by affecting different subsets of proteins within the same PC, interfere with the same biological function, eventually resulting in similar, even if not identical PS.

Finally, to add a further layer of complexity, when predicting the systemic effects of mutations affecting a PC one must take into account that the expression of many proteins could be restricted to certain tissues and cells (such as the rod-restricted mutations in *Retinitis pigmentosa*^27^). As another example, the *Phosphorylase kinase complex*, which catalyzes an activation step in glycogen breakdown, contains either muscle-specific (e.g. PHKA1) or liver-specific (e.g. PHKA2) subunits. These two distinct disease proteins cause clinically different forms of *Glycogen storage disease*, characterized one by muscle weakness and the other by hepatomegaly^28^, so ought not be considered true instances of LH.

In conclusion, our study indicates that there is indeed a substantial intersection of disease proteins and PC in numerous instances of LH, even though the PS-PC relation is far from universal, complete and univocal. However, even with these limitations, our analysis supports the hypothesis that several gene mutations that converge in causing clinically similar LH diseases do in fact represent alternative ways of acting on the same molecular machinery or information-processing unit of the cell. Most often, the affected machinery or unit is one of the PC that have been defined accurately in biochemical terms. Therefore, current biochemical information on the PC in LH is expected to establish important causal links between basic and applied research.

## Methods

### Definition of disease

By ‘disease’, we refer to any item in the Morbid Map of OMIM that has a disease identifier (‘phenotype MIM’), a known molecular basis and is caused by mutations in a protein-coding gene^16^. From the list of diseases (‘phenotypes’) of the Morbid Map, we discarded unconfirmed diseases, non-diseases and susceptibilities to multifactorial disorders (designated with a question mark, square brackets and braces, respectively). Then we also discarded the phenotypes with unknown gene defect, unknown mutation, or caused by multiple genes (designated by the keys 1, 2 and 4, respectively). Second, to map the diseases to the genes, the remaining items in the Morbid Map were merged with the *mim2gene* file of OMIM (after deleting the phenotypes that have been ‘moved’, ‘removed’ or not mapped to Entrez identifiers). Finally, from the disease-gene pairs, we removed the items in which the gene does not code for a protein (e.g., non-coding RNA or pseudogenes).

### Definition of PC

By ‘PC’, we refer to any item in the Cellular Component branch of Gene Ontology^29^ whose name contains the string *complex*. This way, 720 PC were retrieved, annotating 3,265 proteins. Furthermore, as Gene Ontology indicates the hierarchical relationships between its terms (either *PC*_*i*_ *is a PC*_*j*_ or *PC*_*i*_ *is part of PC*_*j*_ format), the PC-PC pairs in a parent-to-child relation were also identified. Aided by these relations, we removed parents of parent PC (unless the child term was devoid of proteins, to avoid loss of information), as well as three generic PC (*Protein complex* GO:0043234, *Transcription factor complex* GO:0005667 and *Macromolecular complex* GO:0032991). In the end 682 PC were left (529 non-parent, 122 parent and 31 parents of parent PC). From these we removed 107 PC that contained only one protein. The remaining 575 PC were used for searching all the instances of LH associated with the PC.

### Instances of LH associated with the PC

By combining the above datasets we generated a list of *3*-tuples, each tuple being composed of one PS, one protein and one PC (as OMIM PS, Entrez Gene and Gene Ontology identifier). Then the table was self-joined to select the pairs of 3-tuples in which two different proteins are components of the same PC and cause diseases belonging to the same PS. The ratio of the PS-PC intersection (the number of proteins shared by both PS and PC) to the PS-PC union (the sum of all the disease proteins in the PS plus all the proteins in the PC) is the JC, which quantifies the PS-PC overlap. A similar strategy was followed for analyzing the disease-based LH, except that the disease (instead of the PS) identifiers were used to assemble the *3*-tuples.

### Human-murine analysis

For each PC, we searched Mouse Genome Informatics^30^ to identify the murine orthologs of the human genes of interest and, where available, the corresponding phenotypes in mice. Murine phenotypes are expressed as terms of the Mammalian Phenotype Ontology^19^. Then, to compare the murine phenotypes (of orthologs of the non-disease proteins) with the human phenotypes (of the disease proteins), the terms of the Murine Phenotype Ontology were translated into the corresponding terms of the Human Phenotype Ontology^31^. Any human non-disease protein with murine-to-human phenotypic resemblance (as defined by the Unified Phenotype Ontology, see ‘Data availability’) was considered a presumptive disease protein and added to the PS-PC intersection, to recalculate the JC. We employed a similar strategy to identify the essential genes.

### Non-univocal PS-PC relations

To identify the non-redundant ‘one-to-many’ PS-PC relations, we discarded all the PS-PC_i_-PC_j_ *3*-tuples in which the two PC are in a parent-child relation, are children of an additional PC, or share more than half their protein content. To identify the ‘many-to-one’ PS-PC relations not due to allele heterogeneity, all the PS-proteins-PC *3*-tuples were paired. Then we discarded the pairs of tuples in which two different PS associate with the same PC but, at the same time, annotate the same protein.

## Supplementary information

Supplementary Information.

Supplementary Information2.

Supplementary Information3.

Supplementary Information4.

## Data Availability

All the datasets used in this manuscript are publicly available from the following sources: OMIM (https://www.omim.org/downloads/) for the gene map and the morbid map. The full list of PS is available from OMIM on request. Gene Ontology (http://geneontology.org/) for the biological features of the PC members. Human Phenotype Ontology (http://www.obofoundry.org/ontology/hp.html) for the disease phenotypes. Mouse Genome Informatics (http://www.informatics.jax.org/downloads/reports/index.html) for the murine orthologs of the human genes and the corresponding phenotypes in mice. Mammalian Phenotype Ontology (http://www.obofoundry.org/ontology/mp.html) for the hierarchical relations among the murine phenotypes. Unified Phenotype Ontology (https://github.com/obophenotype/upheno/) for the mappings of human and mammalian phenotypes (in the hp-to-mp-bestmatches file). The key data generated during the analysis of these datasets are included in this article and its supplementary datasheets. Other analytical data generated during the current study are available from the corresponding author on request. The scripts are available on GitHub at the link https://github.com/alessio-gamba/PC_LH_analysis.
