# Supplementary material for: Quantitative analysis of proteins which are members of the same protein complex but cause locus heterogeneity in disease
Source: Sci Rep. 2020 Jun 26;10:10423. doi: 10.1038/s41598-020-66836-7 (PMC7320193; doi:10.1038/s41598-020-66836-7)
Supplement: Supplementary file 1 — Supplementary Information. [file 41598_2020_66836_MOESM1_ESM.pdf]

Supplementary Information

‘***Quantitative analysis of proteins which are members of the same protein complex but cause locus heterogeneity in disease***’ by Gamba A, Salmona M and Bazzoni G (Istituto di Ricerche Farmacologiche Mario Negri IRCCS, Milano, Italy)

Legends to the Supplementary datasets

Supplementary Table S1. *PS-PC intersections.* The table reports all the PS-PC pairs with a non-null intersection, together with the count of disease proteins in the PS (PS), the count of proteins in the PC (PC), the size of the PS-PC intersection (PS/PC Hs) and the JC (JC Hs). The table also reports (*in red*) the human non-disease proteins in the PC that have a murine ortholog whose mutation causes a similar phenotype to the one caused by the disease proteins (non-disease proteins in Hs), as well as the adjusted size of the intersection (PS/PC Hs + Mm) and the adjusted JC (JC Hs + Mm) after including the murine orthologs in the intersection. The rows are sorted in decreasing order of the adjusted JC. The table reports the counts of disease proteins with annotations of gain-of-function mutations (GOF/PS; see Table S2) and of essential non-disease proteins (Lethal; see Table S3). The acronyms *Hs* and *Mm* indicate *Homo sapiens* and *Mus musculus*.

Supplementary Table S2. *Gain-of-function mutations and LH.* The table reports all the PS-PC pairs in which at least one of the disease proteins contains the words ‘*gain of function*’ (‘GOF’) in the OMIM description of the disease. Proteins in *square brackets* indicate the disease proteins that are also members of the PC.

Supplementary Table S3. *Essential genes in the PC.* The table reports the PC in which at least one protein has a murine ortholog that causes pre-birth lethality in mice. Proteins in *square brackets* indicate murine orthologs of a human protein causing a disease in a PS.
