# Supplementary material for: Quantitative analysis of proteins which are members of the same protein complex but cause locus heterogeneity in disease
Source: Sci Rep. 2020 Jun 26;10:10423. doi: 10.1038/s41598-020-66836-7 (PMC7320193; doi:10.1038/s41598-020-66836-7)
Supplement: Supplementary file 2 — Supplementary Information2. [file 41598_2020_66836_MOESM2_ESM.pdf]

PS name (OMIM id)	PC name (GO id)	Disease proteins in Hs	Non-disease proteins in Hs	PS	PC	PS/PC Hs	JC Hs	*PS/PC Hs+Mm*	*JC Hs+Mm*	Phenotypes (HPO ids)	GOF/PS	Lethal
Methylcrotonylglycinuria (PS210200)	3-methylcrotonyl-CoA carboxylase complex, mitochondrial (GO:0002169)	MCCC1; MCCC2		2	2	2	1	0	1		0	0
Methylcrotonylglycinuria (PS210200)	methylcrotonoyl-CoA carboxylase complex (GO:1905202)	MCCC1; MCCC2		2	2	2	1	0	1		0	0
Paragangliomas (PS168000)	mitochondrial respiratory chain complex II, succinate dehydrogenase complex (ubiquinone) (GO:0005749)	SDHA; SDHB; SDHC; SDHD		5	4	4	0,8	0	0,8		0	0
Epilepsy, familial focal, with variable foci (PS604364)	Iml1 complex (GO:1990130)	DEPDC5; NPRL2; NPRL3		3	4	3	0,75	0	0,75		0	0
Cerebroretinal microangiopathy with calcifications and cysts (PS612199)	CST complex (GO:1990879)	CTC1; STN1		2	3	2	0,667	0	0,667		0	0
Molybdenum cofactor deficiency (PS252150)	molybdopterin synthase complex (GO:0019008)	MOCS1; MOCS2		3	2	2	0,667	0	0,667		0	0
Trichohepatoenteric syndrome (PS222470)	Ski complex (GO:0055087)	SKIV2L; TTC37		2	3	2	0,667	0	0,667		0	0
Tuberous sclerosis (PS191100)	TSC1-TSC2 complex (GO:0033596)	TSC1; TSC2		2	3	2	0,667	0	0,667		0	0
Maple syrup urine disease (PS248600)	mitochondrial alpha-ketoglutarate dehydrogenase complex (GO:0005947)	BCKDHA; BCKDHB; DBT	BCKDK	4	4	3	0,6	1	1	Abnormality of amino acid metabolism; Abnormality of higher mental function (HP:0004337; HP:0011446)	0	0
Three M syndrome (PS273750)	3M complex (GO:1990393)	CCDC8; CUL7; OBSL1	FBXW8	3	5	3	0,6	1	1	Decreased body weight (HP:0004325)	0	1
Bronchiectasis (PS211400)	sodium channel complex (GO:0034706)	SCNN1A; SCNN1B; SCNN1G		3	5	3	0,6	0	0,6		0	0
Acne inversa (PS142690)	gamma-secretase complex (GO:0070765)	NCSTN; PSEN1; PSENEN		3	6	3	0,5	0	0,5		0	2
Polycystic kidney disease (PS173900)	polycystin complex (GO:0002133)	PKD1; PKD2		4	2	2	0,5	0	0,5		0	0
Cold-induced sweating syndrome (PS272430)	CRLF-CLCF1 complex (GO:0097058)	CLCF1; CRLF1		3	3	2	0,5	0	0,5		0	0
Pyruvate dehydrogenase complex deficiency (PS312170)	pyruvate dehydrogenase complex (GO:0045254)	DLAT; PDHA1; PDHB; PDHX		6	6	4	0,5	0	0,5		0	1
Meckel syndrome (PS249000)	MKS complex (GO:0036038)	B9D2; CC2D2A; CEP290; MKS1; TMEM216; TMEM231; TMEM67		9	12	7	0,5	0	0,5		0	2
Cornelia de Lange syndrome (PS122470)	cohesin complex (GO:0008278)	RAD21; SMC1A; SMC3		5	5	3	0,429	0	0,429		0	2
Aicardi-Goutieres syndrome (PS225750)	ribonuclease H2 complex (GO:0032299)	RNASEH2A; RNASEH2B; RNASEH2C		7	3	3	0,429	0	0,429		2	0
Dyskeratosis congenita (PS127550)	box H/ACA snoRNP complex (GO:0031429)	DKC1; NHP2; NOP10		7	4	3	0,375	0	0,375		0	0
Dyskeratosis congenita (PS127550)	box H/ACA scaRNP complex (GO:0072589)	DKC1; NHP2; NOP10		7	4	3	0,375	0	0,375		0	0
Dyskeratosis congenita (PS127550)	box H/ACA telomerase RNP complex (GO:0090661)	DKC1; NHP2; NOP10		7	4	3	0,375	0	0,375		0	0
Joubert syndrome (PS213300)	MKS complex (GO:0036038)	AHI1; B9D1; CC2D2A; CEP290; MKS1; TCTN1; TCTN2; TMEM216; TMEM231; TMEM67		25	12	10	0,37	0	0,37		0	0
Coffin-Siris syndrome (PS135900)	nBAF complex (GO:0071565)	ARID1A; ARID1B; SMARCA4; SMARCB1; SMARCE1	SMARCC1	5	14	5	0,357	1	0,462	Abnormality of skin morphology (HP:0011121)	1	1
Granulomatous disease, chronic (PS306400)	NADPH oxidase complex (GO:0043020)	CYBA; CYBB; NCF1; NCF2	NCF4; NOX4	4	12	4	0,333	2	0,6	Recurrent infections (HP:0002719)	0	1
Coffin-Siris syndrome (PS135900)	SWI/SNF complex (GO:0016514)	ARID1A; ARID1B; SMARCA4; SMARCB1; SMARCE1	RB1; SMARCC1	5	15	5	0,333	2	0,538	Abnormality of skin morphology (HP:0011121)	1	3
Hermansky-Pudlak syndrome (PS203300)	BLOC-2 complex (GO:0031084)	HPS3; HPS5; HPS6		9	3	3	0,333	0	0,333		0	0
Meier-Gorlin syndrome (PS224690)	origin recognition complex (GO:0000808)	ORC1; ORC4; ORC6		6	6	3	0,333	0	0,333		1	0
Fanconi anemia (PS227650)	Fanconi anaemia nuclear complex (GO:0043240)	FANCA; FANCB; FANCC; FANCE; FANCF; FANCG; FANCL	FANCM; FAAP20	16	13	7	0,318	2	0,45	Abnormality of reproductive system physiology; Hypogonadism; Abnormal genital system morphology (HP:0000080; HP:0000135; HP:0012243)	0	0
Hemochromatosis (PS235200)	HFE-transferrin receptor complex (GO:1990712)	HFE; HFE2; TFR2	TF; TFRC	5	8	3	0,3	2	0,625	Abnormality of cation homeostasis (HP:0010929)	1	2
Trichothiodystrophy (PS601675)	core TFIIH complex (GO:0000439)	ERCC2; ERCC3; GTF2H5		5	8	3	0,3	0	0,3		0	0

Hs, Homo sapiens; Mm, Mus musculus; GOF, Gain-of-Function

PS name (OMIM id)	PC name (GO id)	Disease proteins in Hs	Non-disease proteins in Hs	PS	PC	PS/PC Hs	JC Hs	*PS/PC Hs+Mm*	*JC Hs+Mm*	Phenotypes (HPO ids)	GOF/PS	Lethal
Agammaglobulinemia (PS601495)	B cell receptor complex (GO:0019815)	CD79A; CD79B	SYK	6	3	2	0,286	1	0,5	Abnormality of leukocytes; Abnormality of lymphocytes; Abnormality of humoral immunity; Abnormality of B cell physiology (HP:0001881; HP:0004332; HP:0005368; HP:0005372)	0	0
Adams-Oliver syndrome (PS100300)	MAML1-RBP-Jkappa- ICN1 complex (GO:0002193)	NOTCH1; RBPJ		6	3	2	0,286	0	0,286		1	0
Loeys-Dietz syndrome (PS609192)	transforming growth factor beta receptor complex (GO:0070022)	TGFBR1; TGFBR2		5	4	2	0,286	0	0,286		0	2
Colorectal cancer, hereditary nonpolyposis (PS120435)	MutSalpha complex (GO:0032301)	MSH2; MSH6		7	2	2	0,286	0	0,286		0	0
Congenital disorders of glycosylation, type II (PS212066)	Golgi transport complex (GO:0017119)	COG1; COG4; COG5; COG6; COG7; COG8		16	12	6	0,273	0	0,273		0	1
Microcephaly and chorioretinopathy (PS251270)	gamma-tubulin ring complex (GO:0008274)	TUBGCP4; TUBGCP6		3	7	2	0,25	0	0,25		0	1
Progressive external ophthalmoplegia with mtDNA deletions (PS157640)	gamma DNA polymerase complex (GO:0005760)	DNA2; POLG		8	2	2	0,25	0	0,25		0	0
Atrial fibrillation, familial (PS608583)	voltage-gated sodium channel complex (GO:0001518)	SCN1B; SCN2B; SCN3B; SCN4B; SCN5A		12	14	5	0,238	0	0,238		5	0
Osteogenesis imperfecta (PS166200)	macromolecular complex (GO:0032991)	CRTAP; P3H1; PPIB		13	3	3	0,231	0	0,231		0	0
Pontocerebellar hypoplasia (PS607596)	tRNA-intron endonuclease complex (GO:0000214)	CLP1; TSEN2; TSEN54		12	4	3	0,231	0	0,231		1	0
Kabuki syndrome (PS147920)	MLL3/4 complex (GO:0044666)	KDM6A; KMT2D	PAXIP1	2	9	2	0,222	1	0,375	Decreased body weight; Postnatal growth retardation; Abnormality of skin morphology (HP:0004325; HP:0008897; HP:0011121)	0	4
Pigmented nodular adrenocortical disease, primary (PS610489)	cAMP-dependent protein kinase complex (GO:0005952)	PRKACA; PRKAR1A		4	7	2	0,222	0	0,222		1	0
Cowden disease (PS158350)	mitochondrial respiratory chain complex II, succinate dehydrogenase complex (ubiquinone) (GO:0005749)	SDHB; SDHD		7	4	2	0,222	0	0,222		0	2
Coenzyme Q10 deficiency, primary (PS607426)	transferase complex (GO:1990234)	PDSS1; PDSS2		7	5	2	0,2	0	0,2		0	1
Familial restrictive cardiomyopathy (PS115210)	troponin complex (GO:0005861)	TNNI3; TNNT2		4	8	2	0,2	0	0,2		0	0
Ventricular tachycardia, catecholaminergic polymorphic (PS604772)	junctional membrane complex (GO:0030314)	CASQ2; TRDN		5	7	2	0,2	0	0,2		0	2
Aortic aneurysm, familial thoracic (PS607086)	transforming growth factor beta receptor complex (GO:0070022)	TGFBR1; TGFBR2		8	4	2	0,2	0	0,2		1	2
Epilepsy, generalized, with febrile seizures plus (PS604233)	voltage-gated sodium channel complex (GO:0001518)	SCN1A; SCN1B; SCN9A		5	14	3	0,188	0	0,188		0	1
Alternating hemiplegia of childhood (PS104290)	sodium:potassium-exchanging ATPase complex (GO:0005890)	ATP1A2; ATP1A3	ATP1B2	2	11	2	0,182	1	0,3	Morphological abnormality of the central nervous system; Abnormality of movement (HP:0002011; HP:0100022)	0	0
Emery-Dreifuss muscular dystrophy (PS310300)	LINC complex (GO:0034993)	SYNE1; SYNE2	SUN1	6	8	2	0,167	1	0,273	Abnormality of muscle size (HP:0030236)	1	0
Epilepsy, nocturnal frontal lobe (PS600513)	acetylcholine-gated channel complex (GO:0005892)	CHRNA2; CHRNA4; CHRNB2	CHRNB3	4	17	3	0,167	1	0,235	Behavioral abnormality (HP:0000708)	1	1
Usher syndrome (PS276900)	USH2 complex (GO:1990696)	PDZD7; USH2A		12	2	2	0,167	0	0,167		0	0
Muscular dystrophy, limb-girdle, autosomal recessive (PS253600)	dystrophin-associated glycoprotein complex (GO:0016010)	DAG1; FKRP; SGCA; SGCB; SGCD	UTRN; SSPN	21	16	5	0,156	2	0,233	Abnormality of central motor function (HP:0011442)	0	1
Brugada syndrome (PS601144)	L-type voltage-gated calcium channel complex (GO:1990454)	CACNA1C; CACNB2	CACNA1D	9	6	2	0,154	1	0,25	Arrhythmia (HP:0011675)	1	0
Peroxisome biogenesis disorder (PS214100)	peroxisomal importomer complex (GO:1990429)	PEX12; PEX14		13	2	2	0,154	0	0,154		0	0

Hs, Homo sapiens; Mm, Mus musculus; GOF, Gain-of-Function

PS name (OMIM id)	PC name (GO id)	Disease proteins in Hs	Non-disease proteins in Hs	PS	PC	PS/PC Hs	JC Hs	*PS/PC Hs+Mm*	*JC Hs+Mm*	Phenotypes (HPO ids)	GOF/PS	Lethal
Congenital disorders of glycosylation, type I (PS212065)	dolichol-phosphate-mannose synthase complex (GO:0033185)	DPM1; DPM2; DPM3		20	3	3	0,15	0	0,15		1	0
Hermansky-Pudlak syndrome (PS203300)	BLOC-1 complex (GO:0031083)	BLOC1S3; BLOC1S6; DTNBP1	SNAP25; BLOC1S4; PI4K2A	9	15	3	0,143	3	0,333	Morphological abnormality of the central nervous system (HP:0002011)	0	2
Treacher Collins syndrome (PS154500)	DNA-directed RNA polymerase I complex (GO:0005736)	POLR1C; POLR1D		3	13	2	0,143	0	0,143		0	2
Arthrogryposis, renal dysfunction, and cholestasis (PS208085)	HOPS complex (GO:0030897)	VIPAS39; VPS33B		2	14	2	0,143	0	0,143		0	1
Cerebrooculofacioskeletal syndrome (PS214150)	holo TFIIH complex (GO:0005675)	ERCC2; ERCC5	MNAT1	4	13	2	0,133	1	0,214	Abnormality of skin morphology (HP:0011121)	0	3
Leukodystrophy, hypomyelinating (PS312080)	aminoacyl-tRNA synthetase multienzyme complex (GO:0017101)	AIMP1; RARS		13	4	2	0,133	0	0,133		1	2
Seizures, benign familial infantile (PS601764)	voltage-gated sodium channel complex (GO:0001518)	SCN2A; SCN8A		3	14	2	0,133	0	0,133		1	1
Familial episodic pain syndrome (PS615040)	voltage-gated sodium channel complex (GO:0001518)	SCN10A; SCN11A		3	14	2	0,133	0	0,133		3	1
Myasthenic syndrome, congenital (PS601462)	acetylcholine-gated channel complex (GO:0005892)	CHRNA1; CHRNB1; CHRND; CHRNE	CHRNA3; CHRNA7; CHRNG	18	17	4	0,129	3	0,25	Abdominal symptom (HP:0011458)	1	1
Hereditary sensory and autonomic neuropathy (PS162400)	serine C-palmitoyltransferase complex (GO:0017059)	SPTLC1; SPTLC2		13	5	2	0,125	0	0,125		2	1
Seizures, familial febrile (PS121210)	voltage-gated sodium channel complex (GO:0001518)	SCN1A; SCN9A		4	14	2	0,125	0	0,125		0	1
Rubinstein-Taybi syndrome (PS180849)	histone acetyltransferase complex (GO:0000123)	CREBBP; EP300	KAT7	2	20	2	0,1	1	0,158	Abnormality of the face; Decreased body weight; Abnormality of the systemic arterial tree; Abnormal appendicular skeleton morphology; Morphological abnormality of the gastrointestinal tract; Vascular neoplasm (HP:0000271; HP:0004325; HP:0011004; HP:0011844; HP:0012718; HP:0100742)	0	4
Cardiomyopathy, familial hypertrophic (PS192600)	troponin complex (GO:0005861)	TNNC1; TNNI3; TNNT2		25	8	3	0,1	0	0,1		0	0
Muscular dystrophy-dystroglycanopathy, type C (PS609308)	dystrophin-associated glycoprotein complex (GO:0016010)	DAG1; FKRP	UTRN; SSPN	8	16	2	0,091	2	0,2	Abnormality of central motor function (HP:0011442)	0	1
Long QT syndrome (PS192500)	voltage-gated potassium channel complex (GO:0008076)	CALM1; CALM2; KCNE1; KCNE2; KCNH2; KCNJ2; KCNJ5; KCNQ1	HCN2; DLG2; DLG4; KCNJ6; KCNMA1; SNAP25; CNTNAP2; KCNV1	14	88	8	0,085	8	0,186	Morphological abnormality of the central nervous system (HP:0002011)	2	7
Bleeding disorder, platelet-type (PS231200)	integrin complex (GO:0008305)	ITGA2B; ITGB3; MYH9	BST1; ITGAD; ITGAE; ITGAL; ITGAM; ITGB2; ITGB7	16	25	3	0,079	7	0,323	Abnormality of leukocytes (HP:0001881)	1	5
Dilated cardiomyopathy (PS115200)	troponin complex (GO:0005861)	TNNC1; TNNI3; TNNT2		33	8	3	0,079	0	0,079		0	0
Mental retardation, autosomal dominant (PS156200)	nBAF complex (GO:0071565)	ARID1A; ARID1B; SMARCA4; SMARCB1	SMARCC1	43	14	4	0,075	1	0,096	Abnormality of skin morphology (HP:0011121)	4	2
Mental retardation, autosomal dominant (PS156200)	SWI/SNF complex (GO:0016514)	ARID1A; ARID1B; SMARCA4; SMARCB1	RB1; SMARCC1	43	15	4	0,074	2	0,115	Abnormality of skin morphology (HP:0011121)	4	4
Colorectal cancer, hereditary nonpolyposis (PS120435)	synaptonemal complex (GO:0000795)	MLH1; MLH3	MSH5; SYCP1; FKBP6; SYCP2; CCNB1IP1; HORMAD1; HORMAD2; RNF212	7	23	2	0,071	8	0,5	Abnormal genital system morphology (HP:0012243)	0	3
Muscular dystrophy-dystroglycanopathy, type A (PS236670)	dystrophin-associated glycoprotein complex (GO:0016010)	DAG1; FKRP	UTRN; SSPN	14	16	2	0,071	2	0,154	Abnormality of central motor function (HP:0011442)	0	1
Episodic ataxia (PS160120)	voltage-gated calcium channel complex (GO:0005891)	CACNA1A; CACNB4	CACNA1B; CACNA1S; CACNB1; CACNA2D2; CACNA2D3	4	29	2	0,065	5	0,269	Abnormality of central motor function; Abnormality of movement (HP:0011442; HP:0100022)	0	3
Dilated cardiomyopathy (PS115200)	muscle myosin complex (GO:0005859)	MYH6; MYH7; TTN	MYH1; MYH4; MYLPF	33	16	3	0,065	3	0,14	Muscle weakness (HP:0001324)	0	1

Hs, Homo sapiens; Mm, Mus musculus; GOF, Gain-of-Function

PS name (OMIM id)	PC name (GO id)	Disease proteins in Hs	Non-disease proteins in Hs	PS	PC	PS/PC Hs	JC Hs	*PS/PC Hs+Mm*	*JC Hs+Mm*	Phenotypes (HPO ids)	GOF/PS	Lethal
Deafness, autosomal dominant (PS124900)	connexin complex (GO:0005922)	GJB2; GJB3; GJB6	GJC3	29	20	3	0,065	1	0,089	Abnormality of the face; Abnormal peripheral nervous system morphology (HP:0000271; HP:0000759)	5	3
Spermatogenic failure (PS258150)	synaptonemal complex (GO:0000795)	SYCP3; TEX11	HSPA2; MSH5; SYCP1; FKBP6; SYCP2; STAG3; CCNB1IP1; HORMAD1; HORMAD2; RNF212	12	23	2	0,061	10	0,522	Abnormality of reproductive system physiology (HP:0000080)	0	3
Ventricular septal defect (PS614429)	RNA polymerase II transcription factor complex (GO:0090575)	GATA4; NKX2-5	HIF1A; HMGA1; RXRA; HAND1	3	32	2	0,061	4	0,207	Abnormal heart morphology (HP:0001627)	0	10
Retinitis pigmentosa (PS268000)	U4/U6 x U5 tri-snRNP complex (GO:0046540)	PRPF3; PRPF31; PRPF4; PRPF6		57	20	4	0,055	0	0,055		1	1
Atrial septal defect (PS108800)	RNA polymerase II transcription factor complex (GO:0090575)	GATA4; NKX2-5	HIF1A; HMGA1; RXRA; HAND1	8	32	2	0,053	4	0,176	Abnormal heart morphology (HP:0001627)	1	10
Lissencephaly (PS607432)	microtubule associated complex (GO:0005875)	DCX; PAFAH1B1	EML1; MAP1A; MAP1B; MAP2; CLIP2; LRP8; PEA15; KIF1B	9	32	2	0,051	8	0,323	Morphological abnormality of the central nervous system (HP:0002011)	0	7
Epileptic encephalopathy, early infantile (PS308350)	voltage-gated sodium channel complex (GO:0001518)	SCN1A; SCN2A; SCN8A	SCN3A	48	14	3	0,051	1	0,069	Abnormality of higher mental function (HP:0011446)	9	1
Deafness, autosomal recessive (PS220290)	connexin complex (GO:0005922)	GJB2; GJB3; GJB6	GJA10	50	20	3	0,045	1	0,061	Abnormality of vision; Abnormal nervous system electrophysiology; Abnormality of the globe (HP:0000504; HP:0001311; HP:0012374)	0	3
Mental retardation, X-linked syndromic (PS309510)	MLL1 complex (GO:0071339)	LAS1L; TAF1	KMT2A; SENP3	22	28	2	0,042	2	0,087	Behavioral abnormality (HP:0000708)	1	14
Cataract (PS116200)	connexin complex (GO:0005922)	GJA3; GJA8	GJA10	30	20	2	0,042	1	0,064	Abnormality of the face; Abnormality of vision; Abnormality of the globe (HP:0000271; HP:0000504; HP:0012374)	0	5
Nemaline myopathy (PS161800)	Cul3-RING ubiquitin ligase complex (GO:0031463)	KBTBD13; KLHL40; KLHL41	GAN; KEAP1; KBTBD2	11	67	3	0,04	3	0,083	Decreased body weight (HP:0004325)	1	5
Spinocerebellar ataxia (PS164400)	voltage-gated calcium channel complex (GO:0005891)	CACNA1A; CACNA1G	CACNA1B; CACNA1S; CACNB1; CACNA2D2; CACNA2D3	24	29	2	0,039	5	0,152	Abnormality of central motor function; Abnormality of movement (HP:0011442; HP:0100022)	5	3
Spastic paraplegia (PS303350)	AP-type membrane coat adaptor complex (GO:0030119)	AP4M1; AP5Z1		44	9	2	0,039	0	0,039		1	1
Bartter syndrome (PS601678)	chloride channel complex (GO:0034707)	CLCNKA; CLCNKB	CLCN1; GABRA3; GABRA5; GABRB2; GLRA1	6	49	2	0,038	5	0,146	Behavioral abnormality (HP:0000708)	0	2
Parkinson disease (PS168600)	mitochondrial respiratory chain complex I (GO:0005747)	PARK7; SNCA	NDUFS1; NDUFS4	11	43	2	0,038	2	0,08	Decreased body weight; Abnormality of higher mental function (HP:0004325; HP:0011446)	0	4
Cortical dysplasia, complex, with other brain malformations (PS614039)	kinesin complex (GO:0005871)	KIF2A; KIF5C	YWHAE; KIF20B; KIF14; KIF1B; DISC1; NDE1; KIF27; KIF19	6	51	2	0,036	8	0,213	Morphological abnormality of the central nervous system (HP:0002011)	0	11
Short QT syndrome (PS609620)	voltage-gated potassium channel complex (GO:0008076)	KCNH2; KCNJ2; KCNQ1	KCNJ3	3	88	3	0,034	1	0,046	Abnormal EKG; Arrhythmia (HP:0003115; HP:0011675)	1	7
Epileptic encephalopathy, early infantile (PS308350)	voltage-gated potassium channel complex (GO:0008076)	KCNA2; KCNB1; KCNQ2; KCNT1	HCN2; KCNC2; KCNJ3; KCNJ6; KCNMA1; SNAP25; STX1A; KCNAB2; CNTNAP2; KCNV1; KCNIP3	48	88	4	0,03	11	0,124	Abnormality of movement (HP:0100022)	9	7
Pseudohypoaldosteronism, type II (PS145260)	Cul3-RING ubiquitin ligase complex (GO:0031463)	CUL3; KLHL3	RNF7	4	67	2	0,029	1	0,044	Abnormality of cation homeostasis; Abnormality of chloride homeostasis (HP:0010929; HP:0011422)	0	4

Hs, Homo sapiens; Mm, Mus musculus; GOF, Gain-of-Function

PS name (OMIM id)	PC name (GO id)	Disease proteins in Hs	Non-disease proteins in Hs	PS	PC	PS/PC Hs	JC Hs	*PS/PC Hs+Mm*	*JC Hs+Mm*	Phenotypes (HPO ids)	GOF/PS	Lethal
Parkinson disease (PS168600)	ubiquitin ligase complex (GO:0000151)	FBXO7; PARK2; PINK1	BMI1; BRCA1; DNAJA1; LMO7; NEDD4; MED1; TRAF2; RNF8; STUB1; ARIH2; UBR2; FBXL12; FBXO11; MED30; UBR3; MIB2; UBR1	11	104	3	0,027	17	0,211	Decreased body weight (HP:0004325)	0	23
Deafness, autosomal recessive (PS220290)	myosin complex (GO:0016459)	MYO15A; MYO3A	MYH10; MYO10; MYO18A	50	33	2	0,025	3	0,064	Abnormality of the globe (HP:0012374)	0	6
Seizures, benign familial neonatal (PS121200)	voltage-gated potassium channel complex (GO:0008076)	KCNQ2; KCNQ3	HCN2; KCNC2; KCNJ3; KCNJ6; KCNMA1; SNAP25; STX1A; KCNAB2; CNTNAP2; KCNV1; KCNIP3	2	88	2	0,023	11	0,169	Abnormality of movement (HP:0100022)	0	6
Jervell and Lange-Nielsen syndrome (PS220400)	voltage-gated potassium channel complex (GO:0008076)	KCNE1; KCNQ1	KCNMA1; SNAP25	2	88	2	0,023	2	0,047	Hearing impairment (HP:0000365)	0	8
Spastic paraplegia (PS303350)	kinesin complex (GO:0005871)	KIF1A; KIF5A	YWHAE; KIF20B; KIF14; KIF1B; DISC1; NDE1; KIF27; KIF19	44	51	2	0,022	8	0,118	Morphological abnormality of the central nervous system (HP:0002011)	1	10
Hyperinsulinemia hypoglycemia (PS256450)	voltage-gated potassium channel complex (GO:0008076)	ABCC8; KCNJ11	HCN2; KCNA4; KCNC2; KCNJ6; SNAP25; AKAP9; CNTNAP2	7	88	2	0,022	7	0,105	Seizures (HP:0001250)	2	8
Charcot-Marie-Tooth disease (PS118220)	proteasome complex (GO:0000502)	HSPB1; VCP	UBE3A; USP14; UBE3C	42	58	2	0,02	3	0,053	Behavioral abnormality; Morphological abnormality of the central nervous system; Abnormality of movement (HP:0000708; HP:0002011; HP:0100022)	6	6
Diamond-Blackfan anemia (PS105650)	intracellular ribonucleoprotein complex (GO:0030529)	RPL5; RPS7	BRCA1; EPRS; ERG; FMR1; NPM1; YBX1; G3BP1; IGF2BP1; LRRK2; JAKMIP1	10	116	2	0,016	10	0,105	Morphological abnormality of the central nervous system (HP:0002011)	0	26
Amyotrophic lateral sclerosis (PS105400)	intracellular ribonucleoprotein complex (GO:0030529)	ATXN2; HNRNPA1	BRCA1; EPRS; ERG; FMR1; NPM1; YBX1; G3BP1; IGF2BP1; LRRK2; JAKMIP1	23	116	2	0,015	10	0,094	Morphological abnormality of the central nervous system (HP:0002011)	3	24

Hs, Homo sapiens; Mm, Mus musculus; GOF, Gain-of-Function
