# Supplementary material for: Quantitative analysis of proteins which are members of the same protein complex but cause locus heterogeneity in disease
Source: Sci Rep. 2020 Jun 26;10:10423. doi: 10.1038/s41598-020-66836-7 (PMC7320193; doi:10.1038/s41598-020-66836-7)
Supplement: Supplementary file 3 — Supplementary Information3. [file 41598_2020_66836_MOESM3_ESM.pdf]

PS name (OMIM id)	PC name (GO id)	Disease Proteins GOF	Disease Proteins all	GOF-annotated Proteins [PC members]
Adams-Oliver syndrome (PS100300)	MAML1-RBP-Jkappa- ICN1 complex (GO:0002193)	1	6	ARHGAP31
Aicardi-Goutieres syndrome (PS225750)	ribonuclease H2 complex (GO:0032299)	2	7	IFIH1; TREX1
Amyotrophic lateral sclerosis (PS105400)	intracellular ribonucleoprotein complex (GO:0030529)	3	23	C9orf72; FUS; SOD1
Aortic aneurysm, familial thoracic (PS607086)	transforming growth factor beta receptor complex (GO:0070022)	1	8	PRKG1
Atrial fibrillation, familial (PS608583)	voltage-gated sodium channel complex (GO:0001518)	5	12	KCNA5; KCNE2; KCNJ2; KCNQ1; NPPA
Atrial septal defect (PS108800)	RNA polymerase II transcription factor complex (GO:0090575)	1	8	TBX20
Bleeding disorder, platelet-type (PS231200)	integrin complex (GO:0008305)	1	16	PLAU
Brugada syndrome (PS601144)	L-type voltage-gated calcium channel complex (GO:1990454)	1	9	KCND3
Charcot-Marie-Tooth disease (PS118220)	proteasome complex (GO:0000502)	6	42	LITAF; NEFH; NEFL; PMP22; SH3TC2; TRPV4
Coffin-Siris syndrome (PS135900)	SWI/SNF complex (GO:0016514)	1	5	[SMARCB1]
	nBAF complex (GO:0071565)			[SMARCB1]
Congenital disorders of glycosylation, type I (PS212065)	dolichol-phosphate-mannose synthase complex (GO:0033185)	1	20	ALG13
Deafness, autosomal dominant (PS124900)	connexin complex (GO:0005922)	5	29	COCH; DFNA5; DIAPH1; TBC1D24; TMC1
Emery-Dreifuss muscular dystrophy (PS310300)	LINC complex (GO:0034993)	1	6	LMNA
Epilepsy, nocturnal frontal lobe (PS600513)	acetylcholine-gated channel complex (GO:0005892)	1	4	[CHRNA4]
Epileptic encephalopathy, early infantile (PS308350)	voltage-gated potassium channel complex (GO:0008076)	9	48	ALG13; GNAO1; GRIN2B; GRIN2D; HCN1; [KCNA2]; [KCNT1]; SCN8A; SLC1A2
	voltage-gated sodium channel complex (GO:0001518)			ALG13; GNAO1; GRIN2B; GRIN2D; HCN1; KCNA2; KCNT1; [SCN8A]; SLC1A2
Familial cold autoinflammatory syndrome (PS120100)		2	3	NLRP12; PLCG2
Familial episodic pain syndrome (PS615040)	voltage-gated sodium channel complex (GO:0001518)	3	3	[SCN10A]; [SCN11A]; TRPA1
Hemochromatosis (PS235200)	HFE-transferrin receptor complex (GO:1990712)	1	5	[HFE]
Hereditary sensory and autonomic neuropathy (PS162400)	serine C-palmitoyltransferase complex (GO:0017059)	2	13	SCN11A; [SPTLC1]
Hyperinsulinemia hypoglycemia (PS256450)	voltage-gated potassium channel complex (GO:0008076)	2	7	GLUD1; SLC16A1
Hypocalcemia (PS601198)		2	2	CASR; GNA11
Leukodystrophy, hypomyelinating (PS312080)	aminoacyl-tRNA synthetase multienzyme complex (GO:0017101)	1	13	PLP1
Long QT syndrome (PS192500)	voltage-gated potassium channel complex (GO:0008076)	2	14	CACNA1C; CAV3
Meier-Gorlin syndrome (PS224690)	origin recognition complex (GO:0000808)	1	6	GMNN
Mental retardation, X-linked syndromic (PS309510)	MLL1 complex (GO:0071339)	1	22	PRPS1
Mental retardation, autosomal dominant (PS156200)	SWI/SNF complex (GO:0016514)	4	43	GNB1; GRIN1; GRIN2B; [SMARCB1]
	nBAF complex (GO:0071565)			GNB1; GRIN1; GRIN2B; [SMARCB1]
Myasthenic syndrome, congenital (PS601462)	acetylcholine-gated channel complex (GO:0005892)	1	18	[CHRNB1]
Myopathy, tubular aggregate (PS160565)		2	2	ORAI1; STIM1
Nemaline myopathy (PS161800)	Cul3-RING ubiquitin ligase complex (GO:0031463)	1	11	TPM3
Neurodegeneration with brain iron accumulation (PS234200)		2	6	FTL; PLA2G6
Noonan syndrome (PS163950)		6	9	NRAS; PTPN11; RAF1; RIT1; SOS1; SOS2
Pigmented nodular adrenocortical disease, primary (PS610489)	cAMP-dependent protein kinase complex (GO:0005952)	1	4	[PRKACA]
Pontocerebellar hypoplasia (PS607596)	tRNA-intron endonuclease complex (GO:0000214)	1	12	KCNT1
Retinitis pigmentosa (PS268000)	U4/U6 x U5 tri-snRNP complex (GO:0046540)	1	57	NR2E3
Seizures, benign familial infantile (PS601764)	voltage-gated sodium channel complex (GO:0001518)	1	3	[SCN8A]
Short QT syndrome (PS609620)	voltage-gated potassium channel complex (GO:0008076)	1	3	[KCNH2]
Spastic paraplegia (PS303350)	AP-type membrane coat adaptor complex (GO:0030119)	1	44	BSCL2
	kinesin complex (GO:0005871)			BSCL2
Spinocerebellar ataxia (PS164400)	voltage-gated calcium channel complex (GO:0005891)	5	24	ATXN1; ATXN3; ATXN8; [CACNA1A]; NOP56
Thrombophilia (PS188050)		2	9	F2; F9
