# Supplementary material for: Quantitative analysis of proteins which are members of the same protein complex but cause locus heterogeneity in disease
Source: Sci Rep. 2020 Jun 26;10:10423. doi: 10.1038/s41598-020-66836-7 (PMC7320193; doi:10.1038/s41598-020-66836-7)
Supplement: Supplementary file 4 — Supplementary Information4. [file 41598_2020_66836_MOESM4_ESM.pdf]

PC name (GO id)	Genes lethal	Genes all	Orthologs of human PC proteins causing pre-birth lethality in mouse [Disease proteins in PS]
3M complex (GO:1990393)	2	5	[Cul7]; Fbxw8
acetylcholine-gated channel complex (GO:0005892)	1	17	Stxbp5
aminoacyl-tRNA synthetase multienzyme complex (GO:0017101)	2	4	Eprs; [Kars]
AMPA glutamate receptor complex (GO:0032281)	1	27	Porcn
AP-type membrane coat adaptor complex (GO:0030119)	1	9	Ap1g1
BLOC-1 complex (GO:0031083)	2	15	Bloc1s1; Bloc1s2
calcium channel complex (GO:0034704)	4	24	Mcu; [Pkd1l1]; Ryr1; [Ryr2]
cAMP-dependent protein kinase complex (GO:0005952)	1	7	[Prkar1a]
chloride channel complex (GO:0034707)	2	49	[Gabrg2]; Ttyh1
clathrin adaptor complex (GO:0030131)	3	12	Ap1g1; Ap1m1; Gga2
cohesin complex (GO:0008278)	4	5	Cdca5; [Rad21]; [Smc3]; Wapl
connexin complex (GO:0005922)	5	20	[Gja1]; [Gjb2]; [Gjb3]; Gjb5; Gjc1
core TFIIH complex (GO:0000439)	2	8	[Ercc2]; [Ercc3]
Cul3-RING ubiquitin ligase complex (GO:0031463)	5	67	[Cul3]; Glmn; Kctd10; Rbx1; Rnf7
DNA-directed RNA polymerase I complex (GO:0005736)	2	13	Polr1b; Polr2h
DNA-directed RNA polymerase III complex (GO:0005666)	1	18	Polr2h
dolichol-phosphate-mannose synthase complex (GO:0033185)	2	3	[Dpm1]; [Dpm3]
dystrophin-associated glycoprotein complex (GO:0016010)	3	16	[Dag1]; [Fkrp]; Krt8
ERCC4-ERCC1 complex (GO:0070522)	3	4	[Ercc1]; [Slx4]; Xrcc1
Fanconi anaemia nuclear complex (GO:0043240)	1	13	[Fancl]
GABA-A receptor complex (GO:1902711)	1	18	[Gabrg2]
gamma DNA polymerase complex (GO:0005760)	2	2	[Dna2]; [Polg]
gamma-secretase complex (GO:0070765)	4	6	Aph1a; [Ncstn]; [Psen1]; Tmed10
gamma-tubulin ring complex (GO:0008274)	1	7	Brca1
Golgi transport complex (GO:0017119)	1	12	Tmem115
HFE-transferrin receptor complex (GO:1990712)	2	8	Bmpr1a; Tfrc
histone acetyltransferase complex (GO:0000123)	6	20	[Crebbp]; [Ep300]; [Ikbkap]; Kat7; Kat8; Mcrs1
histone methyltransferase complex (GO:0035097)	16	23	Ash2l; Cxxc1; Dpy30; Jarid2; [Kdm6a]; Kmt2a; [Kmt2b]; Kmt2c; [Kmt2d]; [Men1]; Ncoa6; Paxip1; Prmt5; Setd1a; Setd1b; Zfp335
Holliday junction resolvase complex (GO:0048476)	2	5	[Rad51c]; [Slx4]
holo TFIIH complex (GO:0005675)	4	13	Cdk7; [Ercc2]; [Ercc3]; Mnat1
HOPS complex (GO:0030897)	1	14	Vps41
Iml1 complex (GO:1990130)	1	4	[Nprl3]
integrin complex (GO:0008305)	6	25	Itga5; Itga7; Itgav; Itgb1; Itgb8; [Myh9]
intracellular ribonucleoprotein complex (GO:0030529)	26	116	[Actb]; Ago2; [Atxn2]; Brca1; Cpsf3; Dhx9; [Dyrk1a]; Eprs; Erg; Gapdh; Gsk3b; Gtf3c1; [Hnrnpa1]; Hnrnpl; Hnrnpu; Ilf2; Jmjd6; Larp7; Lrpprc; Npm1; Nsrp1; [Nup62]; Secisbp2; Ssb; Ybx1; Zfp36l1
junctional membrane complex (GO:0030314)	2	7	[Jph2]; Ryr1
kinesin complex (GO:0005871)	11	51	Cenpe; Kif11; Kif16b; Kif18a; [Kif1a]; Kif1b; Kif20b; Kif22; Kif5b; Ndel1; [Pafah1b1]
L-type voltage-gated calcium channel complex (GO:1990454)	2	6	[Cacna1c]; [Cacnb2]
MAML1-RBP-Jkappa- ICN1 complex (GO:0002193)	2	3	[Notch1]; [Rbpj]
meiotic cohesin complex (GO:0030893)	2	6	Rec8; [Smc3]
microtubule associated complex (GO:0005875)	8	32	[Dnaaf2]; Fnta; Fntb; Kif1b; Map1b; [Pafah1b1]; Pxn; Ranbp2

PC name (GO id)	Genes lethal	Genes all	Orthologs of human PC proteins causing pre-birth lethality in mouse [Disease proteins in PS]
mitochondrial respiratory chain complex I (GO:0005747)	4	43	Ndufa13; Ndufa5; Ndufs7; Ndufv2
mitochondrial respiratory chain complex II, succinate dehydrogenase complex (ubiquinone) (GO:0005749)	4	4	[Sdha]; [Sdhb]; [Sdhc]; [Sdhd]
MKS complex (GO:0036038)	6	12	[B9d1]; [Cc2d2a]; [Cep290]; [Mks1]; [Tctn1]; [Tmem231]
MLL1 complex (GO:0071339)	14	28	Ash2l; Chd8; Kat8; Kmt2a; Max; Mcrs1; Mga; [Prpf31]; Rnf2; Ruvbl1; Taf4; Taf6; Taf7; Tex10
MLL3/4 complex (GO:0044666)	6	9	Ash2l; Dpy30; [Kdm6a]; Kmt2c; [Kmt2d]; Paxip1
muscle myosin complex (GO:0005859)	3	16	[Myh6]; Myl1; [Ttn]
myosin complex (GO:0016459)	6	33	Myh10; [Myh6]; [Myl2]; Myl7; Myo18a; [Myo1e]
myosin II complex (GO:0016460)	2	6	Myh10; [Myh9]
NADPH oxidase complex (GO:0043020)	1	12	Ncf4
nBAF complex (GO:0071565)	5	14	[Arid1a]; [Smarca4]; [Smarcb1]; Smarcc1; [Smarce1]
npBAF complex (GO:0071564)	7	12	Actl6a; [Arid1a]; [Smarca4]; [Smarcb1]; Smarcc1; [Smarce1]; Ss18
nuclear origin of replication recognition complex (GO:0005664)	2	9	Mcm2; [Orc1]
origin recognition complex (GO:0000808)	1	6	[Orc1]
peroxisomal importomer complex (GO:1990429)	1	2	[Pex14]
polycystin complex (GO:0002133)	2	2	[Pkd1]; [Pkd2]
proteasome complex (GO:0000502)	7	58	Psmc1; Psmc3; Psmc4; Psmd4; Psmd7; [Vcp]; [Wfs1]
protein phosphatase type 2A complex (GO:0000159)	4	21	Cycs; Ppp2ca; [Ppp2r1a]; Strn
pyruvate dehydrogenase complex (GO:0045254)	2	6	[Dld]; [Pdha1]
respiratory chain complex II (GO:0045273)	2	2	[Sdhb]; [Sdhc]
RNA polymerase II transcription factor complex (GO:0090575)	12	32	Arnt; Ascl2; Carm1; [Gata4]; Hand1; Hif1a; [Nkx2-5]; Nr5a2; [Pparg]; Rxra; [Stat3]; Trim28
serine C-palmitoyltransferase complex (GO:0017059)	3	5	[Sptlc1]; [Sptlc2]; Sptssa
Ski complex (GO:0055087)	1	3	[Skiv2l]
small nucleolar ribonucleoprotein complex (GO:0005732)	1	9	Naf1
spliceosomal complex (GO:0005681)	16	89	Ctnnbl1; Ddx41; Gemin2; [Hnrnpa1]; Hnrnpc; Lsm4; Ppp1r8; Prpf18; [Prpf3]; Rheb; Sf1; Sf3a1; Sf3b1; [Snrnp200]; Srsf2; Wdr83
SWI/SNF complex (GO:0016514)	7	15	Actl6a; [Arid1a]; Rb1; [Smarca4]; [Smarcb1]; Smarcc1; [Smarce1]
synaptonemal complex (GO:0000795)	3	23	Plk1; Ube2i; Wapl
telomerase holoenzyme complex (GO:0005697)	4	21	Hnrnpc; Hnrnpu; Smg6; [Wrap53]
transcription factor TFIID complex (GO:0005669)	12	35	[Ercc1]; [Ercc2]; [Ercc3]; Gtf2b; Taf10; Taf4; Taf6; Taf7; Taf8; [Tbp]; Tcea1; [Trp53]
transferase complex (GO:1990234)	2	5	[Pdss2]; Ube2i
transforming growth factor beta receptor complex (GO:0070022)	4	4	Dab2; [Eng]; [Tgfbr1]; [Tgfbr2]
tRNA-intron endonuclease complex (GO:0000214)	1	4	[Clp1]
troponin complex (GO:0005861)	1	8	[Tnnt2]
TSC1-TSC2 complex (GO:0033596)	2	3	[Tsc1]; [Tsc2]
U4/U6 x U5 tri-snRNP complex (GO:0046540)	3	20	Prpf18; [Prpf3]; [Prpf31]
ubiquitin ligase complex (GO:0000151)	23	104	Arih2; Bard1; Brap; Brca1; Fbxl5; [Ikbkg]; Med1; Med11; Med12; Med21; Med24; Med31; Mib2; Nedd4; Rnf2; Rnf20; Sugt1; Traf2; Ube2l3; Ube2n; Ube4b; Ubr2; Ubr3
voltage-gated calcium channel complex (GO:0005891)	3	29	[Cacna1c]; Cacna1s; [Cacnb2]
voltage-gated potassium channel complex (GO:0008076)	8	88	Cttn; [Kcna1]; [Kcnh2]; [Kcnq2]; [Kcnq3]; Kcnq5; Stx1a; [Sumo1]
voltage-gated sodium channel complex (GO:0001518)	1	14	[Scn5a]
